# A157 DEFINITIONS OF MEDITERRANEAN DIET INCONSISTENTLY ASSOCIATE WITH MARKERS OF GUT BARRIER FUNCTION OR SUBCLINICAL INFLAMMATION IN A POPULATION-BASED COHORT

**DOI:** 10.1093/jcag/gwab049.156

**Published:** 2022-02-21

**Authors:** A Neustaeter, J Timpano, S Lee, M Xue, H Leibovitzh, K Madsen, J Meddings, O Espin-Garcia, A Goethel, A Griffiths, P Moayyedi, H Steinhart, R Panaccione, H Q Huynh, K Jacobson, G Aumais, D R Mack, C N Bernstein, J Marshall, W Xu, W Turpin, K Croitoru

**Affiliations:** 1 Department of Gastroenterology, Mount Sinai Hospital, Toronto, ON, Canada; 2 University of Alberta, Edmonton, AB, Canada; 3 Medicine, University of Calgary, Calgary, AB, Canada; 4 Immunology, University of Toronto, Faculty of Medicine, Toronto, ON, Canada; 5 Hospital for Sick Children, Toronto, ON, Canada; 6 McMaster University, Hamilton, ON, Canada; 7 BC Children’s Hospital, Vancouver, BC, Canada; 8 Mount Sinai Hospital, Joseph and Wolf Lebovic Health Complex, Zane Cohen Centre for Digestive Diseases, Toronto, ON, Canada; 9 Mount Sinai Hospital, Toronto, ON, Canada; 10 Uiversity of Manitoba, Winnipeg, MB, Canada; 11 McMaster University Medical Centre, Hamilton, ON, Canada; 12 Pediatrics, University of alberta, Edmonton, AB, Canada; 13 Hopital Maisonneuve-Rosemont, Montreal, QC, Canada; 14 Children’s Hospital of Eastern Ontario, Ottawa, ON, Canada

## Abstract

**Background:**

The Mediterranean Diet (MD) is proposed to reduce the risk of Crohn’s disease (CD) onset in cohort studies, with inconsistent results. This inconsistency may be due to heterogeneity in defining MD scores. Additionally, relationships between MD compliance and intestinal permeability or sub-clinical inflammation are not defined.

**Aims:**

We examined correlations between different MD scores, and determined associations between MD compliance and intestinal permeability or subclinical inflammation in a cohort of first degree relatives of CD patients.

**Methods:**

We used food frequency questionnaire data from 2,112 subjects of the Crohn’s Colitis Canada- Genes, Environment, Microbial (CCC-GEM) project. We obtained 12 MD definitions from the literature and calculated daily percent compliance, we further compared MD scores via pairwise correlations (Kendall’s Tau). We measured intestinal permeability via urinary fractional excretion ratio of lactulose to mannitol (LMR) (LMR≥0.03 defined abnormal), and subclinical inflammation via fecal calprotectin (FCP) measured with BÜHLMANN fCAL® ELISA (FCP≥250 defined abnormal). We fit multivariable regression models between MD compliance and abnormal LMR and FCP, respectively. Two-sided p<0.05 defined significance.

**Results:**

There was large variation in cross-correlations among MD scores, from nil (t=0.0, p=0.54) to highly significant (t=0.97, p<2.2e-16). Associations of MD compliance and abnormal LMR or FCP were in both directions of effect, largely non-significant. Of the 12 MD scores, none associated with abnormal LMR, while 4 associated with abnormal FCP-Odds Ratios =1.22, 1.23, 1.24, and 1.30; p=0.02, 0.02, 0.01, and 0.009, and 95% Confidence Intervals = [1.03,1.45], [1.04,1.45], [1.05,1.47], and [1.07,1.59] respectively. No diet remained significant after correcting for multiple testing.

**Conclusions:**

Currently MD definitions vary widely. Despite discrepancies, we expected consistent directions of effect for MD compliance on LMR or FCP. The largely non-significant associations between MDs suggest limitations in definition, interpretation, and relation to biological outcomes.

Submitted on behalf of the CCC-GEM consortium.

**Funding Agencies:**

CIHRCrohn’s and Colitis Canada Genetics Environment Microbial (CCC-GEM) III;The Leona M. and Harry B. Helmsley Charitable Trust; Justine Timpano is a recipient of a fellowship award from Mount Sinai Hospital; Kenneth Croitoru is the recipient of the Canada Research Chair in Inflammatory Bowel Diseases

